# Modification of Cellulose Nanocrystals With 2-Carboxyethyl Acrylate in the Presence of Epoxy Resin for Enhancing its Adhesive Properties

**DOI:** 10.3389/fbioe.2021.797672

**Published:** 2022-01-28

**Authors:** Amjad Ali, Tariq Aziz, Jieyuan Zheng, Fan Hong, Mahamed F. Awad, Sehrish Manan, Fazal Haq, Asmat Ullah, Muhammad Naeem Shah, Qaiser Javed, Ameer Ali Kubar, Li Guo

**Affiliations:** ^1^ Research School of Polymeric Materials, School of Materials Science and Engineering, Jiangsu University, Zhenjiang, China; ^2^ College of Chemical and Biological Engineering, Zhejiang University, Hangzhou, China; ^3^ Department of Biology, College of Science, Taif University, Taif, Saudi Arabia; ^4^ School of the Environment and Safety Engineering, Jiangsu University, Zhenjiang, China; ^5^ Department of Chemistry, Gomal University, Dera Ismail Khan, Pakistan; ^6^ School of Pharmacy, Xi’an Jiaotong University, Xi’an, China; ^7^ College of Electronics and Information Engineering, Shenzhen University, Shenzhen, China; ^8^ State Key Laboratory of Clean Energy Utilization, Zhejiang University, Hangzhou, China

**Keywords:** cellulose nanocrystals, adhesion, modification, mechanical properties, thermal properties

## Abstract

Cellulose nanocrystals (CNCs) have unparalleled advantages in the preparation of nanocomposites for various applications. However, a major challenge associated with CNCs in nanocomposite preparation is the lack of compatibility with hydrophobic polymers. The hydrophobic modification of CNCs has attracted increasing interest in the modern era standing with long challenges and being environmentally friendly. Here, we synthesized CNCs by using cotton as raw material and then modified them with 2-carboxyethyl acrylate to improve their corresponding mechanical, adhesive, contact angle, and thermal properties. Different concentrations (1–5 wt%) of CNCs were used as modifiers to improve the interfacial adhesion between the reinforced CNCs and E-51 (Bisphenol A diglycidyl ether) epoxy resin system. CNCs offered a better modulus of elasticity, a lower coefficient of energy, and thermal expansion. Compared with the standard sample, the modified CNCs (MCNCs) showed high shear stress, high toughness, efficient degradation, thermal stability, and recycling due to the combined effect of the hyperbranched topological structure of epoxy with good compatibility. The native CNCs lost their hydrophilicity after modification with epoxy, and MCNCs showed good hydrophobic behavior (CA = 105 ± 2°). The findings of this study indicate that modification of CNCs with 2-carboxyethyl acrylate in the presence of epoxy resin and the enhancement of the features would further expand their applications to different sectors.

**GRAPHICAL ABSTRACT:**
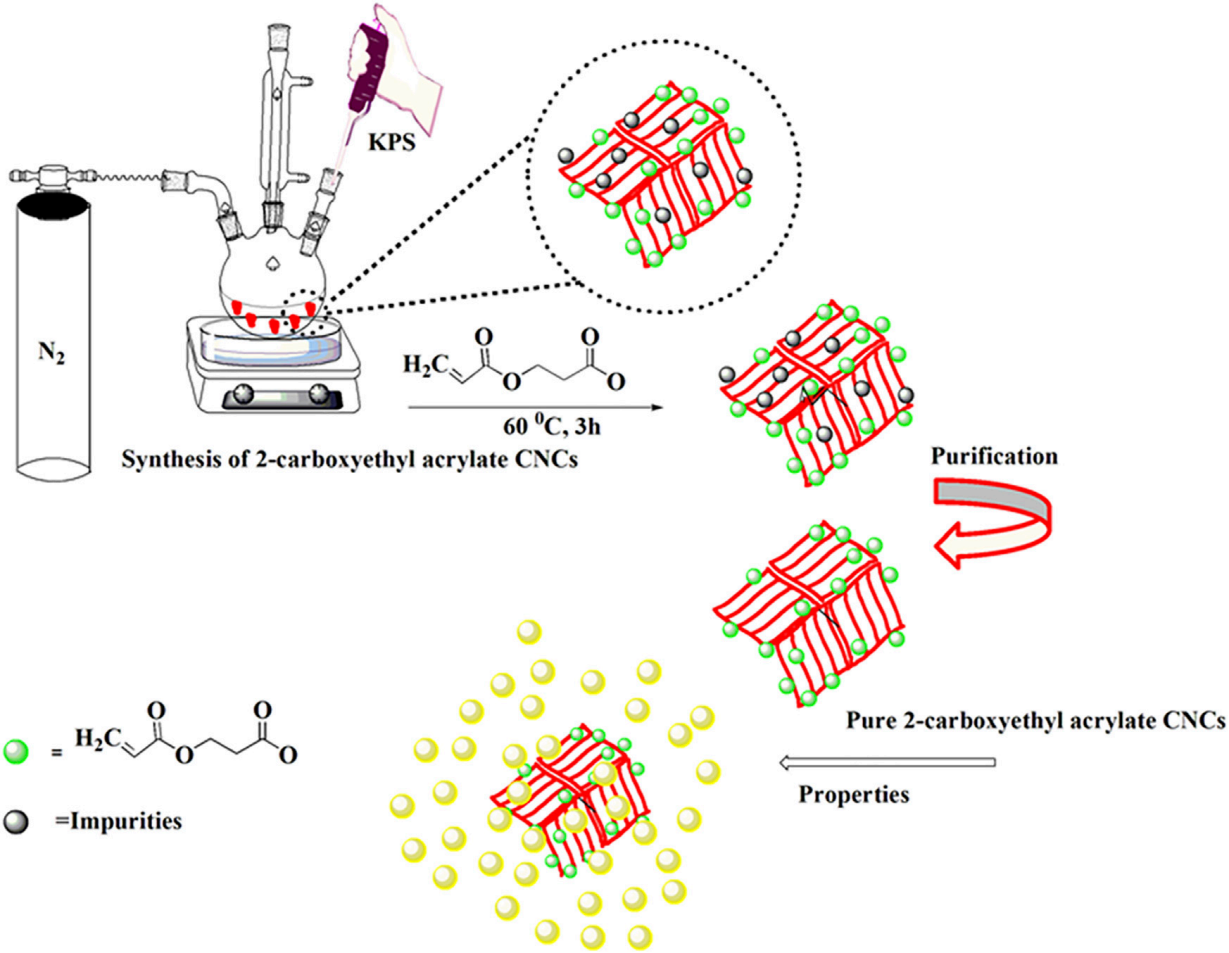


## Introduction

Cellulose nanocrystals (CNCs) or nanoparticles have drawn a lot of attention due to their abundance, biocompatibility, renewability, and excellent mechanical properties, paving the way to innovative and sustainable applications. CNCs are crystalline and rod-shaped, depending on their source. These have been used as reinforcing agents due to their excellent mechanical properties (Dhar et al., 2012; Aziz et al., 2020a; Cao et al., 2020; Satam et al., 2020). These have a large surface area, and their intermolecular structure enables them to interact with other materials (Aziz et al., 2019a; Aziz et al., 2020b; Aziz et al., 2020c). CNCs can form new resin bonds in compounds and improve the mechanical strength of the final product (Guo et al., 2017; Auclair et al., 2020; Aziz et al., 2020d; Rasool et al., 2021). These also form a cluster of relatively small networks and increase the flexibility modulus (Limousin et al., 2020; Popescu et al., 2020; Haq et al., 2021). The cellulose-based aerogels are novel third-generation aerogels that have recently attracted much attention due to their high adsorption efficiency, eco-friendly nature, and cost-effectiveness. Such aerogels acquire several properties, especially with their low cost and chemical stability (Aziz et al., 2021a; Aziz et al., 2021b; Aziz et al., 2021c), and are used as modifiers to enhance the interfacial adhesion between the matrix of nanocrystals (Aziz et al., 2019b; Li et al., 2020; Rincón-Iglesias et al., 2020). Cellulose has a natural nanostructure that allows the impregnation of CNCs. The CNCs have a width of 5–75 nm and a length of more than 100 μm (Pietrzak et al., 2016; Alharthi and El Rassi, 2018; Wijaya et al., 2020). Recently, the uses of CNCs as a modifier have been studied in combination with a wide range of natural or synthetic polymers, especially in an epoxy emulsion (Dastjerdi et al., 2018; Kamtsikakis et al., 2021).

There are several limitations associated with the use of CNCs, mainly caused by their relatively low thermal stability. Therefore, using their hydroxyl surface chemistry to give these nanoparticles a new functionality is very interesting. There are many examples of modifying the CNCs’ synthesis routes (Girouard et al., 2016; Shi et al., 2019; Aziz et al., 2021d). Presently, researchers focused on environmental issues and on providing an affordable and scalable approach for sustainable development have faced one of the most fundamental challenges (Poaty et al., 2014; Li et al., 2018; Alanis et al., 2019; Ullah et al., 2021a). CNCs are a growing area for nanomaterials research candidates because of their attraction for reinforcing agents and due to their reproducibility, high shear stress, and elastic modulus (Yang et al., 2013; Wu et al., 2020; Fang et al., 2021; Niinivaara et al., 2021). CNCs have a high specific surface energy that tends to aggregate during the manufacturing process, forming larger particles. These are also found in powder or an aqueous suspension after purification from amorphous cellulose or other impurities and are used in melt processing applications (Khoshkava and Kamal, 2014; Ali et al., 2020; Seok and Kim, 2020; Akram et al., 2021). Over the past decade, CNCs have been considered a potentially natural nanomaterial and attracted researchers’ attention for their wide applications. These are used as a nanomaterial for various applications due to their excellent physiochemical properties. The scattering and accumulation in polar solvents in a series of hashes attracted optical and structural properties. These characteristics have a long-term effect on the overall performance and relationship.

The 2-Carboxyethyl acrylate is a new auspicious substitute monomer due to its molecular composition and high mechanical properties that can potentially replace the toxic water-soluble monomers. The carboxyl group in 2-carboxyethyl acrylate is expected to positively affect the adhesive behavior of CNCs suspension (Salama et al., 2015; Pietrzak et al., 2016; Alharthi and El Rassi, 2018). The 2-Carboxyethyl acrylate is more hydrophilic than inert monomer and allows its chemical modification (Yang et al., 2020; Rasool et al., 2021; Jamil et al., 2021; Muhammad et al., 2021). It has better physical bonding with metal surfaces or other functional materials and may give better strength to the substrate. Furthermore, as an organic monomer, 2-carboxyethyl acrylate is used with CNCs for enhancing their mechanical strength (Kim et al., 2007; Tripathi et al., 2015; Naseer et al., 2021; Awais et al., 2022).

Herein, we synthesized CNCs using cotton as raw material, and these were further modified with 2-carboxyethyl acrylate to improve their adhesion and thermal properties. CNCs were selected as modifiers to improve the interfacial adhesion in E-51 epoxy resin. The novel strategy used in this study will help prepare regenerated cellulose nano-materials with excellent mechanical properties and biodegradability as alternatives to petrochemical plastics for the development of sustainable materials and could be applied in food packaging.

## Methods and Chemical Reagents

### Materials

Cotton was supplied by the Guangzhou Liqi textile industry (China). 2-Carboxyethyl acrylate was purchased from ChemSrc China (98% purity). Analytical grade ethanol (purity 99.7%), methanol (purity 99.5%), and acetone (purity 99.5%) were obtained from Aladdin (Shanghai, China). Tianjin Hengxing Chemical industry (China) supplied sulfuric acid (purity 98%). Bisphenol-A epoxy resin E-51 for the adhesive property was supplied by Helin Resin Co., Ltd. The commercially available epoxy was used for comparison and purchased from the local market (Jiangsu province, China). Shanghai Macklin Biochemical Co. Ltd. China supplied potassium persulfate (KPS). Triethylenetetramine (TETA) (purity 97%) was obtained from Sigma-Aldrich. Lab-made distilled water was used.

### Preparation of CNCs From Cotton

**FIGURE 1:**
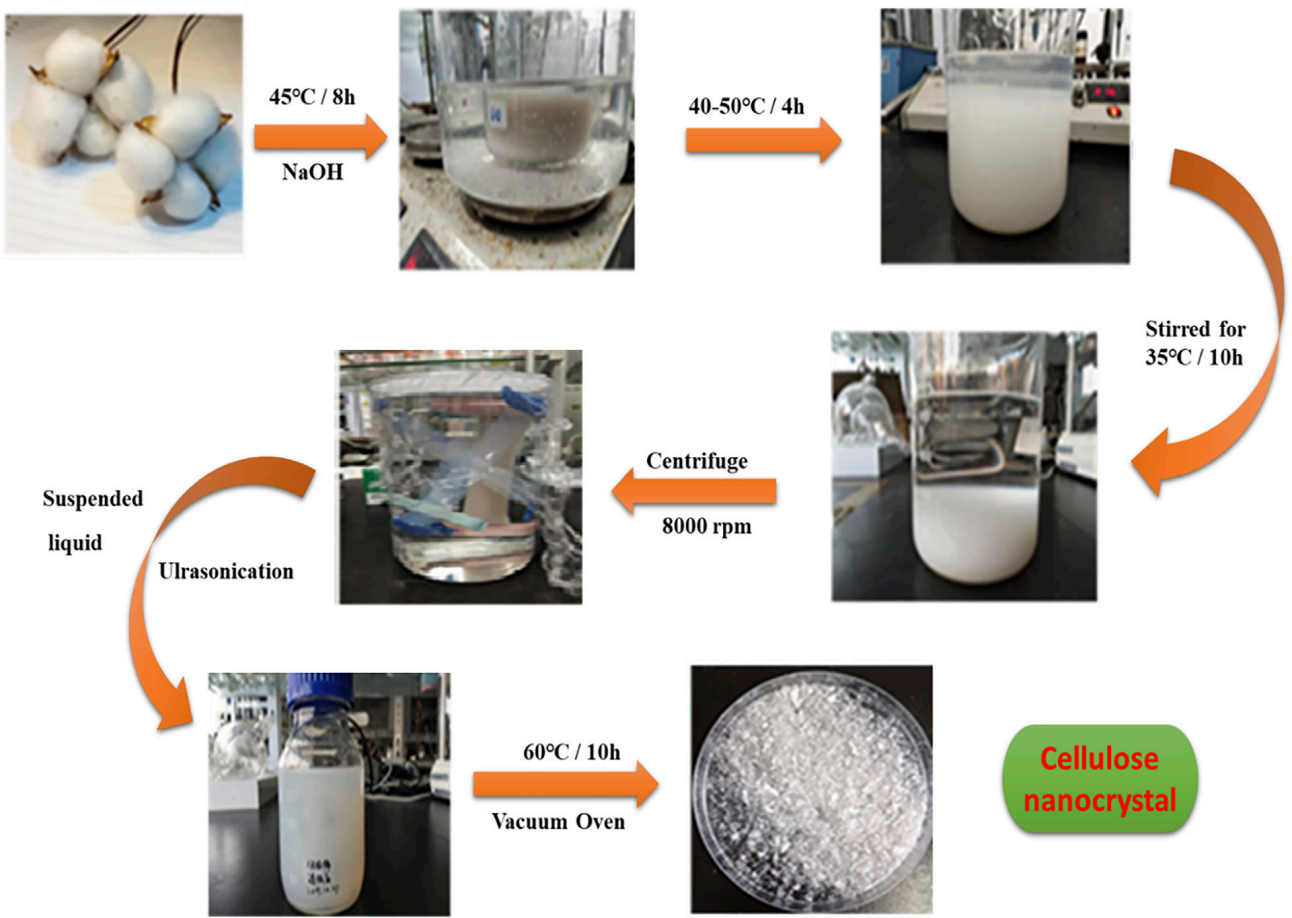
Schematic illustration of the preparation of CNCs from cotton. The alkaline treatment of cotton fibers at elevated temperature, centrifugation, ultrasonication, and vacuum oven drying resulted in the preparation of CNCs.

First, wax and pectin were removed from cotton. A 20-g cotton was cut into small pieces and washed with hot water at 50–60°C. The washed cotton was heated in a hot air oven at 40°C for 6–8 h. Then, the cotton was mixed with 500 ml of 20% NaOH at 45°C for 4–5 h. The alkali-treated cotton slurry was allowed to cool down to room temperature, and the solution was transferred to 2.5 L of distilled water until the pH was neutralized. The neutral suspension was filtered using a Buckner filter. The sample was then heated and stirred in 500 ml of 60% sulfuric acid for 12 h at 35°C. The resulting particles formed a white suspension that was transferred to the 2 L of water to deactivate the white suspension. The suspension was kept statically for 12 h to allow the settling of cellulose particles. After decantation, the white slurry was again treated with distilled water to remove sodium and sulfate ions. The suspension was centrifuged at 8,000 rpm three times for 10 min. The nanocrystals were vigorously shaken at 40°C with 45–60 wt% to remove H_2_SO_4_. The final white product (CNC powder) was dried in a vacuum oven at 90°C for 12 h. A schematic of the preparation of CNCs from cotton is illustrated in Figure 1 **.**

### Modification of CNCs With 2-Carboxyethyl Acrylate

**FIGURE 2:**
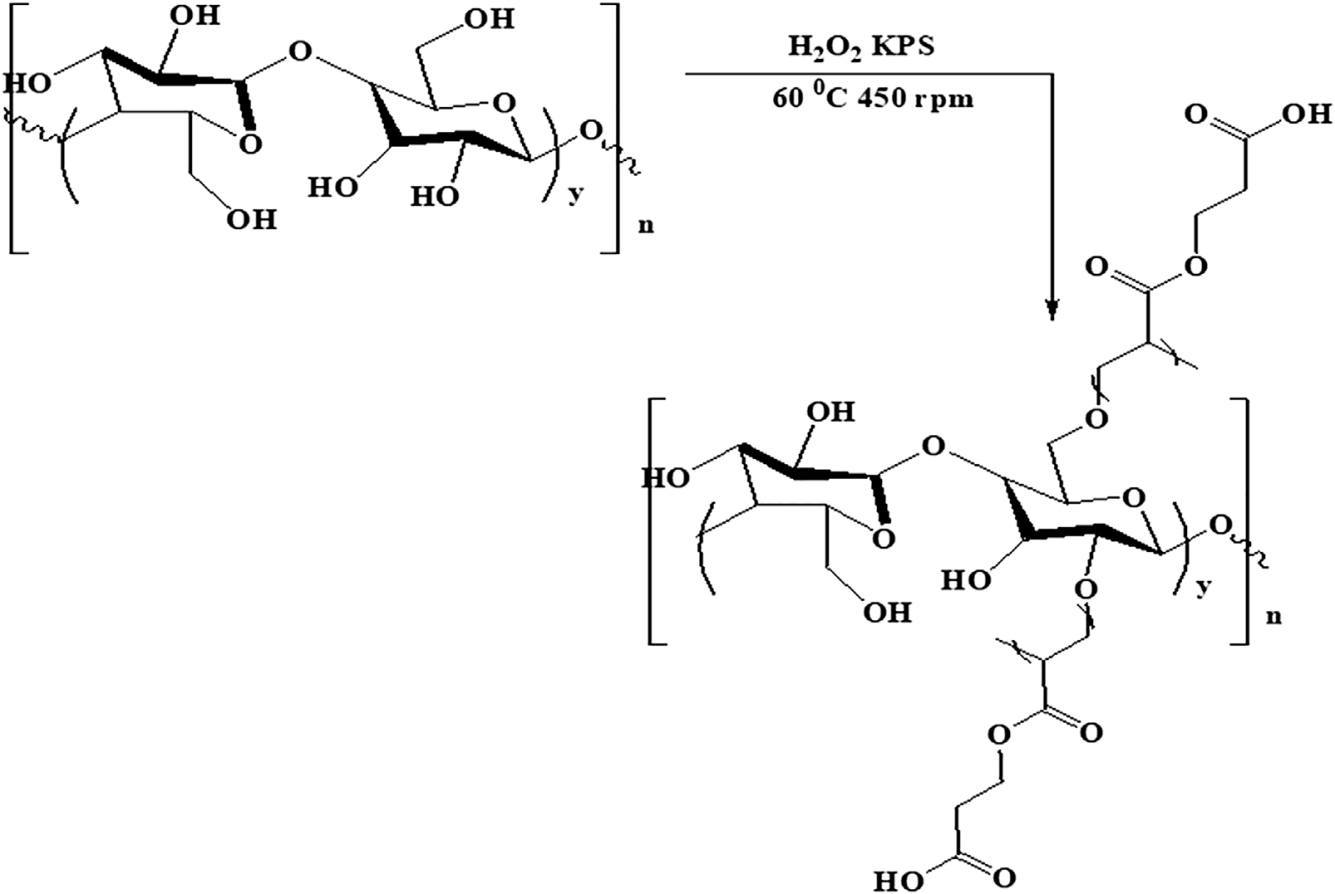
Chemical synthesis of CNCs-*g*-poly (2-carboxyethyl acrylate).

First, CNCs were dispersed in 40 ml of lab-made distilled water at 60°C with stirring for 30 min at room temperature in a nitrogen environment. Then 66.6 mg of potassium persulfate (KPS) was dissolved in 20 ml of distilled water and injected into the solution with the help of a syringe. After 30 min, 2.1 ml of 2-carboxyethyl acrylate monomer was also injected, and the reaction was allowed to stir at 60°C for 3 h. The synthesis of CNCs-*g*-poly (2-carboxyethyl acrylate) is shown in Figure 2. The product was cooled down to room temperature and washed three times with a mixture of 30 ml methanol and 70 ml, and then centrifuged at 5,000 rpm for 10 min. The mixture was washed again with acetone to remove the un-grafted polymers. The process was repeated three times. Then, the product was then placed in the vacuum oven for 24 h at 40°C to completely dry. After that, the sample was put into the bottle and named modified cellulose nanocrystals (MCNCs) and stored in a desiccator for further investigation and characterizations.

### Characterization

FTIR spectroscopy was used to examine the chemical structure of modified CNCs with 2-carboxyethyl acrylate (Nicolet. 5700). The surface morphology of native and modified CNCs was observed through a scanning electron microscope (SEM, Model SU-3500) operated at 20 kV. A transmission electron microscope (TEM, Model Hitachi, Japan) was used for structural analysis of CNCs. Thermogravimetric and DTG properties of 2-carboxyethyl acrylate were investigated under constant N_2_ flow using a TGA analyzing system (TA-Q500, Mettler-Toledo). The crystalline properties of CNCs and MCNCs were investigated *via* an x-ray diffractometer (X’Pert-APD Philips, Netherlands). An ultrasonic cleaner ultrasound Instrument Co., Ltd. was used for sonication of CNCs. The high-speed desktop centrifuge (Cence TG16-WS) was used for the isolation of modified CNCs. For contact angle (Contact angle meter DSA 25) with interfacial and surface tension 0.01–2000 mN/m having resolution 0.01°/0.01 mN/m with illumination high power monochromatic LED. The mechanical and adhesive properties of native and modified CNCs were also determined (Model Zwick/Roel Z020, Germany).

### Adhesive Strength Test

**FIGURE 3:**
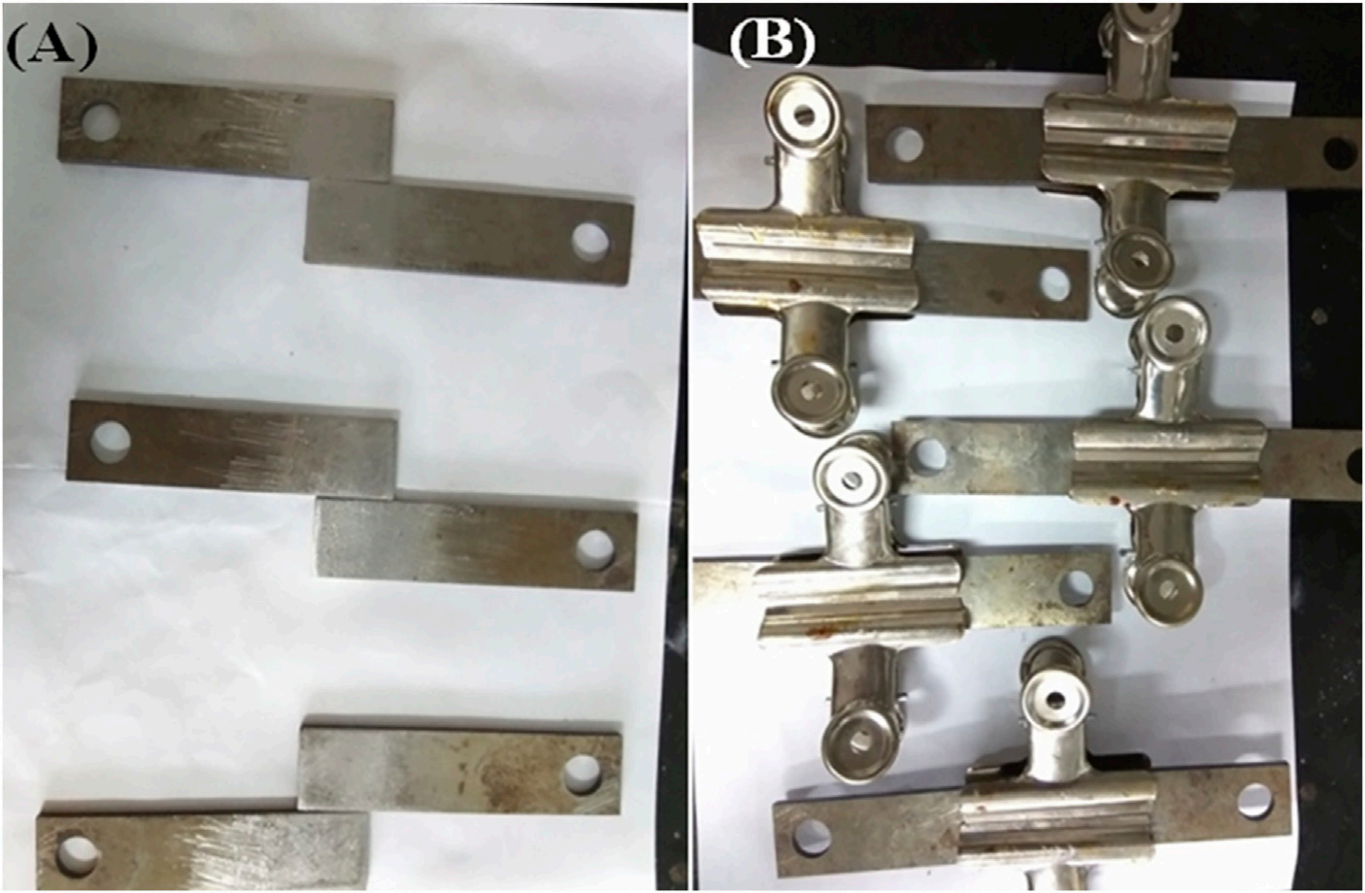
Pictorial representation of **(A)** Polish steel plates of 1.3 cm length and 1.5 cm width and **(B)** steel plates with epoxy and CNCs.

E-51 epoxy resin was placed on the sample glass bottle, and then the CNCs were added. The resultant mixture was sonicated at 45°C for more than 30 min, and then its suspension was prepared by mechanical mixing. The suspension was then circulated through the agitator until all samples of CNCs were thoroughly dispersed in 1 g of E-51 epoxy resin at room temperature with a magnetic bar for adhesive performance. After 1 h of stirring on a hot plate at room temperature, 0.2 g of TETA was added and stirred slowly for the next 20–25 min until a completely homogeneous solution was obtained. The homogeneous solution was stored in a vacuum oven at 45°C for 10–15 min to remove bubbles from the solution. The CNCs and E-51 epoxy resin mixture was poured onto steel plates, as shown in Figure 3, using a 1.5-cm-long and 2.3-cm-wide glass rod. Three different samples with standard one were run for testing in a hot air oven at 150°C for 60 min. The samples were then cooled to room temperature for 3–4 days before testing.

## Results and Discussion

### Morphology of CNCs

**FIGURE 4:**
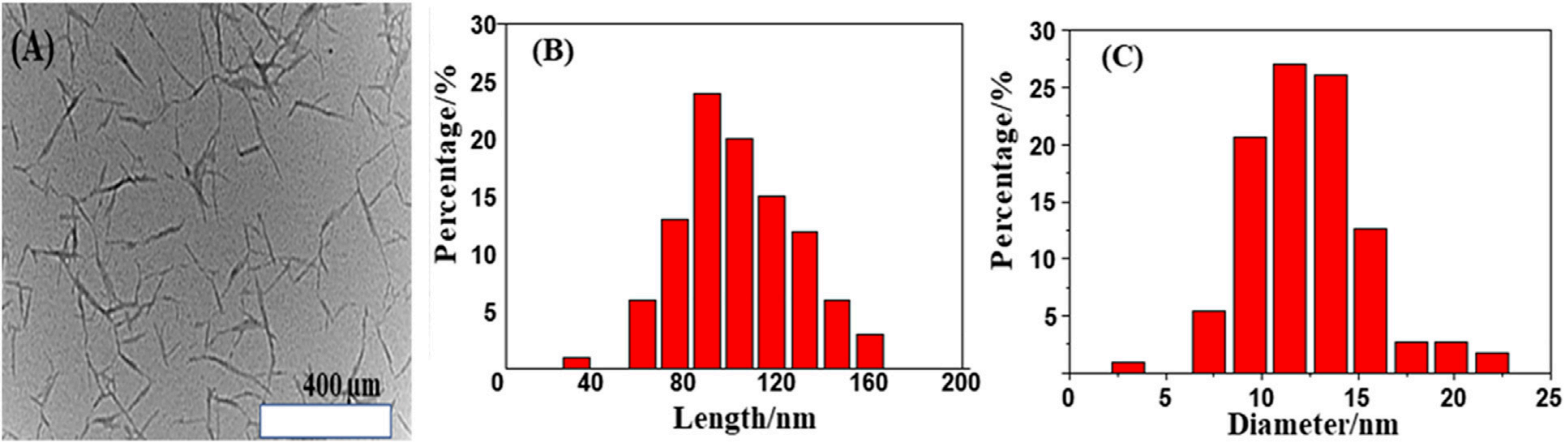
**(A)** The rod-shaped morphology of CNCs observed through TEM, **(B)** length, and **(C)** diameter distribution of CNCs.

A TEM view of the native CNCs produced from cotton through acid hydrolysis is shown in Figure 4A, which shows the typical rod-like morphology. The TEM image shows the presence of a compact, crystal-like, uniform nano-sized rod, which depends on its concentration in the epoxy system (Abraham et al., 2016; Jiang et al., 2020; Qin et al., 2020; Haq et al., 2021). The dimensions of CNCs were also determined. The results show that the mean length of CNCs is 103.47 nm (Figure 4B), and the mean width is 12.31 nm (Figure 4C). Typically, 88.86% of the CNCs with a mean diameter of 8.4 nm were obtained. The remaining crystals exhibited aggregation behavior due to a large number of hydroxyl groups on the surface of the CNCs (Yang et al., 2015; Roeder et al., 2016; Rincón-Iglesias et al., 2020). The lower dose of CNCs showed a cluster-like behavior, which may be higher and improve the interface, which is reflected in the rough surface layer. However, a good dispersion was achieved at a low concentration of CNCs with epoxy resins. A higher concentration of CNCs produced more surface inertia. The obtained CNCs’ shape is rod-like.

### FTIR Analysis of CNCs-*g*-Poly (2-Carboxyethyl Acrylate)

**FIGURE 5:**
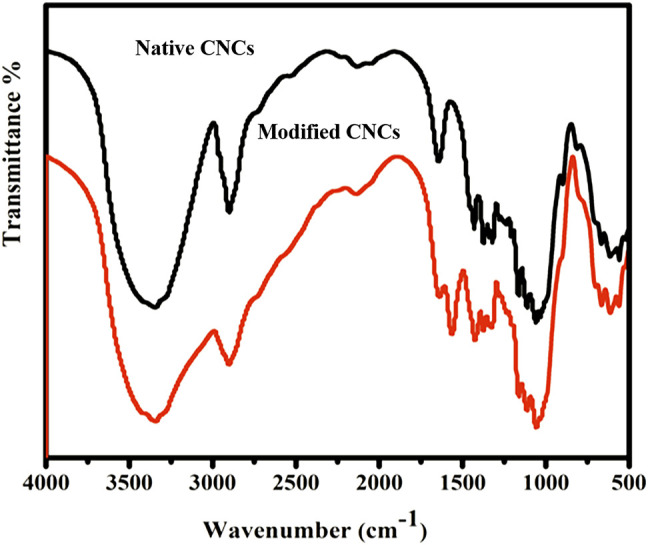
FTIR spectra of native and modified CNCs.

The typical FTIR spectra of native CNCs and MCNCs are shown in Figure 5. A strong peak shown at 755 cm^−1^ was due to the C-H bending (Wulandari et al., 2016; Abdul Rahman et al., 2017). The peak for C-O asymmetric in contrast to the spectrum of MCNCs appeared at 1,030 cm^−1^. A new vibration peak that appeared at 1,750 cm^−1^ was ascribed to the C=O group stretching vibration of the ester and carboxylic group present in MCNCs (Lu et al., 2015; Khan et al., 2018; Aziz et al., 2019c). The appearance of such a peak in MCNCs confirms the successful modification of CNCs. Due to the limitation of size quantification of the CNC particles and its sensitivity towards large particles, the size estimation was used to identify aggregates within different CNCs dispersions. The analysis presented a significant reduction of aggregation in the CNCs-COOH and the modified CNCs with respect to the native one’s dispersion.

### Thermogravimetric Analysis of CNCs

**FIGURE 6:**
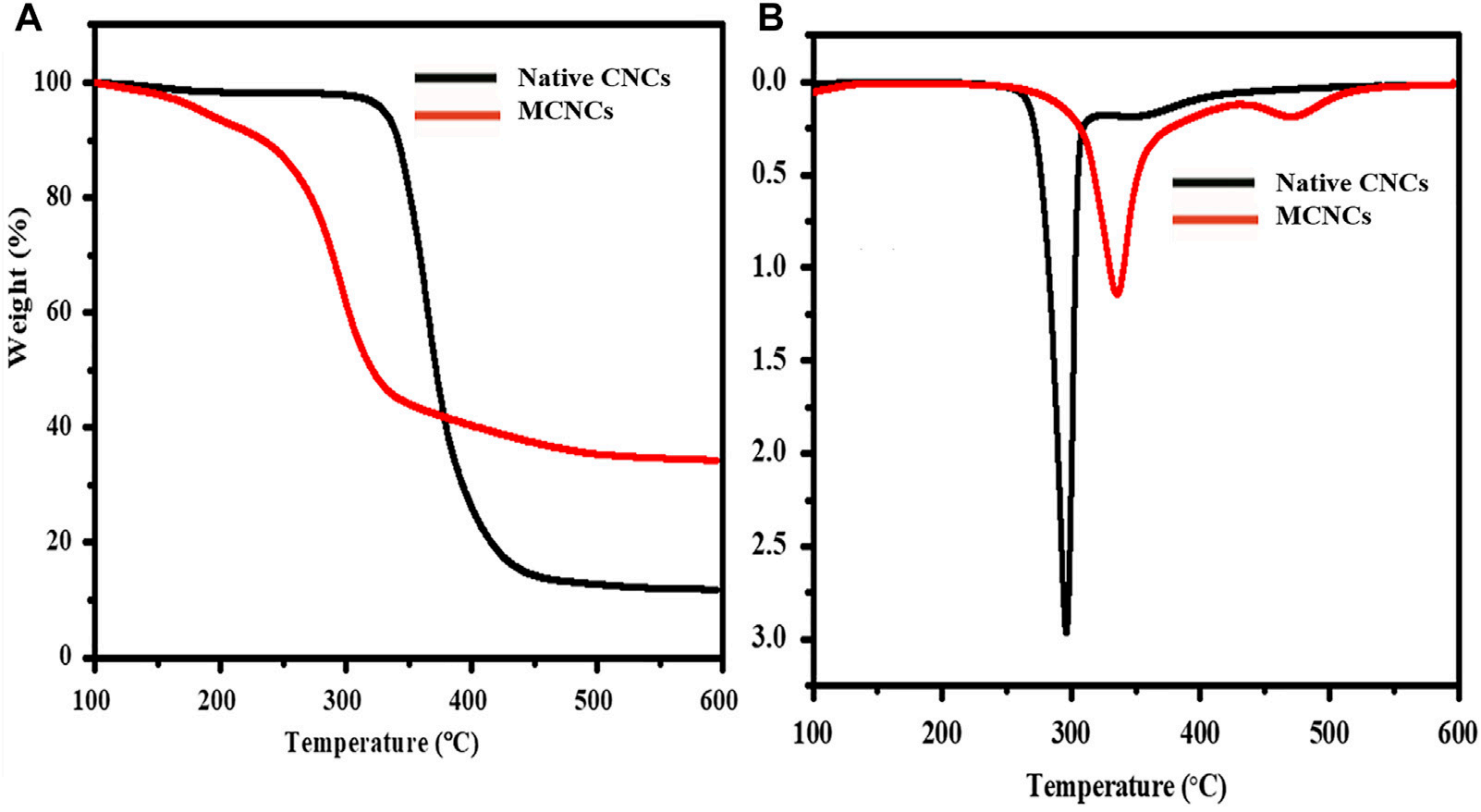
**(A)** TGA and **(B)** DTG curves of native and modified CNCs.

Thermogravimetric analysis (TGA) of native CNCs and MCNCs is a dynamic phenomenological approach to investigate the response to change in temperature. The thermal behavior of native CNCs is different from that of MCNCs, as shown in Figure 6A, which is in accordance with previous reports (Li et al., 2011; Ma et al., 2017). In the case of MCNCs, thermal degradation occurred at a higher temperature due to its nano-size and high thermal stability; more free ends in the MCNCs show a significant decrease in molecular weight degradation in the high amorphous regions. The MCNCs showed a typical decomposition, starting at a temperature above 220–370°C, by introducing silicone groups, leaving a small amount of ash at 600°C, as reported previously (Lu and Hsieh, 2010; Kumar et al., 2014).

The thermal behavior of CNCs showed two stages of thermal degradation, as shown in Figure 6B. The first stage began at about 340°C and is characterized by a direct attachment of the C-C bond structure. The following two measures against deterioration are related to the terminal chain of CH. These degradations occurred at a temperature below the main structure of saturated MCNCs with an E-51 resin system. This behavior is due to the poor stability of the CH and CH_2_ groups, which allow the splitting into β-carbons. Evaluation of the thermal stability of the initial mass loss temperature was determined by the intersection of the initial plated additional mass at the maximum TG curve. The different peaks were first obtained at the maximum temperature. It is only once happening in the process of changing the temperature. The second phase is defined as the temperature that begins and represents the same value for both samples. TGA is an important parameter because of information about the dynamics of mass loss. Unstable emissions during the decomposition process are of great value (Akgün et al., 2016; Souza Neto et al., 2019; Liu et al., 2020). The MCNCs reinforced liquid compound transformation into the poly-condensation with excellent growth after dispersion in the epoxy resin system.

### Morphology of MCNCs

**FIGURE 7:**
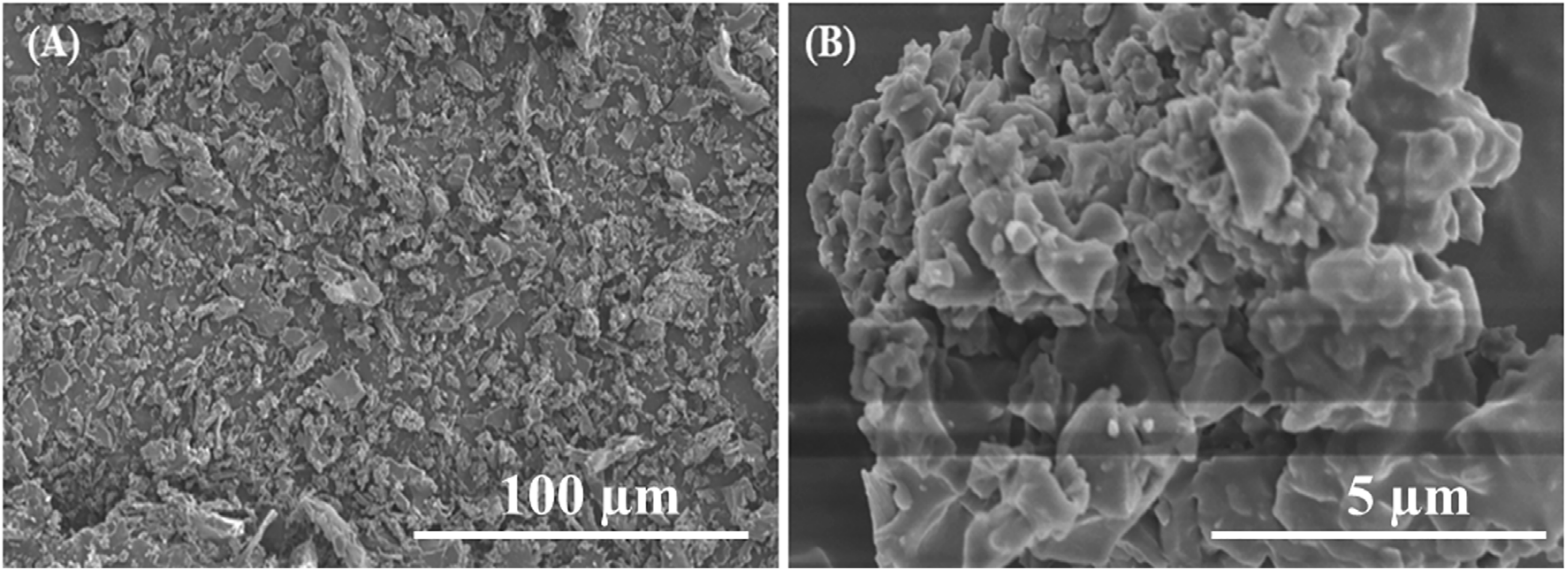
Scanning electron micrographs of freeze-dried **(A)** native and **(B)** modified CNCs.

The SEM images were obtained *via* SEM observation after sputter coating of gold on the surface of the sample. The SEM images of the fracture surface gave a clear indication of CNCs’ handedness. The results also demonstrate that high-resolution SEM of CNCs can appraise the detailed structure system. The apparent porosity in the fracture surfaces reported here may result from the pull-out of nanocrystals to the fracture surface. This information provides a basis for these materials’ exploitation and templating properties. The morphologies of the surface images of dried and modified CNCs were obtained and clearly showed the surface morphologies reacting with the E-51 epoxy system, as shown in Figure 7. The diameters of dried CNCs are decreased after modifying to varying degrees. The surface is much rougher. In general, their combination has a favorable effect on the performance of the composites, which results in increases in the adhesive properties. In the modified CNCs, agglomeration also occurs due to the aggregation of E-51 epoxy, affecting the adhesive properties. The more modified CNCs, the more agglomeration occurs in the epoxy system. The epoxy also affects the adhesive properties when the diamine curing agent (DDS) is used, compared with the TETA. The TETA gives a good result mainly in the dispersion process of CNCs in the epoxy. At this stage, the solution is much thinner, and nanoparticles are easily dispersed in the solution (Du et al., 2017; Ahmed et al., 2021). The presence of pores and cavities in the fractured surfaces decreased significantly compared to the neat ones. The fracture surface does not show much more aggregations of the nanocrystals covered by the epoxy system. The epoxy reinforces the fractured surface of the modified CNCs composite. The coverage of both native CNCs and modified matrix was expected and favored by attractive interactions between polar groups and non-polar domains from both the CNCs and the epoxy, which cause the reinforcement.

### XRD Analysis of Dried and Modified CNCs

**FIGURE 8:**
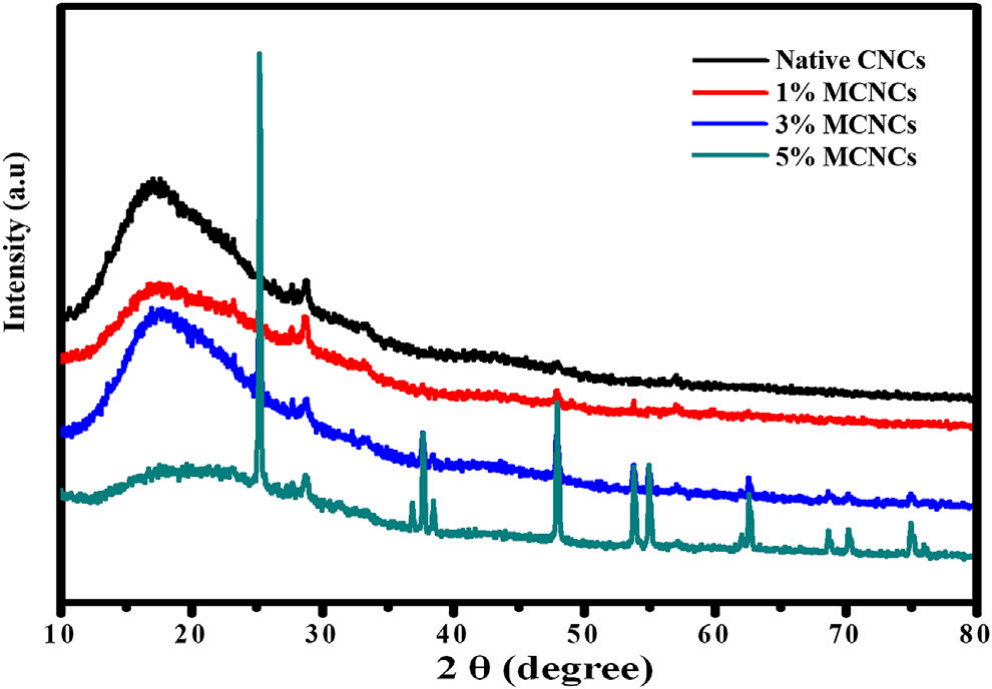
XRD spectra of native and modified CNCs of different concentrations (1%, 3%, and 5%).

In the nanocomposite system, XRD spectra of the composite system are obtained at room temperature and investigate the chemical groups contributing during the curing process. Several bands of XRD spectra conforming to epoxide vibration are at the range 2θ degree = 10 to 20, but here, the epoxide vibration band is at 2θ degree = 25 (existing epoxide ring). This peak intensity directly depends on the absorption of epoxide groups in the mixture of E-51 epoxy resin. The peaks near 2θ degree = 48 and 58 show that the epoxide ring deformation is weaker, which is in accordance with previous reports (Jamil et al., 2018; Jamil et al., 2019). The other peaks of XRD spectra show epoxy resin backing vibrations that do not change the intensity throughout the curing process. In comparison, the starching peak of C-H in the oxirane ring was hardener in spectra, as shown in Figure 8.

### Eugenol-Based Silane Coupling Agent

Eugenol-based silane coupling agent (EBSCA) was synthesized *via* hydrosilylation. This silane coupling agent enhanced the connection between CNCs and epoxy matrix to achieve sustainable and environment-friendly products. A eugenol-based epoxy silane coupling agent with high purity was prepared and used for the surface modification of nano-cellulose crystals. The eugenol epoxy silane-coupling agent, bearing a long-chain structure of benzene ring in the molecular structure, could improve the compatibility of CNCs with the E-51 epoxy system, contributing to the dispersion state in the matrix, enhancing the overall performance of epoxy-cured products.

### Adhesive Properties of Modified CNCs

**FIGURE 9:**
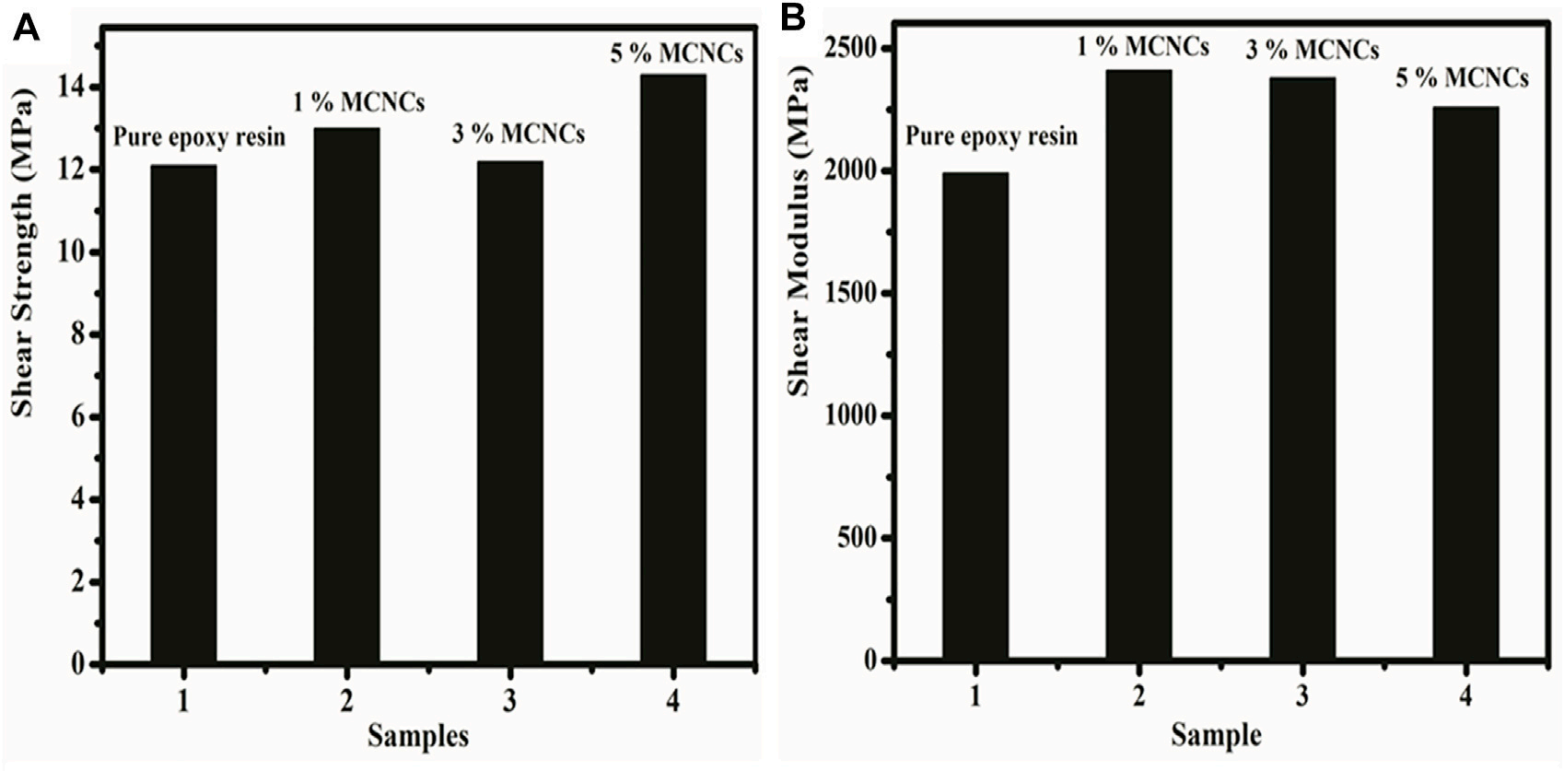
**(A)** Shear strength and **(B)** shear modulus of pure epoxy resins and different concentrations (1%, 3%, and 5%) of MCNCs.

We studied the adhesive properties of MCNCs with an epoxy system by measuring the average shear strength (Figure 9A) and shear modulus (Figure 9B) using the E-51 epoxy resin with different content. TETA was used as a curing agent. The E-51 epoxy system containing MCNCs in an amount of 1%, 3%, and 5 wt% was used to evaluate the effect of adhesive properties. The shear strength showed enhancement at 5 wt% of MCNCs. It could be due to the crystal agglomeration in the epoxy resin medium, making them harder, as reported previously (Al-Turaif, 2013; Ferreira et al., 2014). The maximum value was observed at 15.1 MPa for nanocomposites reinforced with 5 wt% MCNCs compared with the pure epoxy resin, which is in harmony with previous studies (Pinheiro et al., 2017; Ullah et al., 2021b). Compared with the standard sample, all the MCNC samples had a higher shear modulus. The maximum value (2,460 MPa) was observed for 1 wt% modified nanocrystal to compete with the standard value of 1,990 MPa. We believe that the result of MCNCs with E-51 epoxy resin, -OH bond, is responsible for binding themselves. They are creating a high strength linkage and increasing the properties of the adhesive additives. MCNC shear modulus loading (5 wt%) agglomeration and aggression occur between particles in an epoxy system, which cause weakness, as reported previously (Dastjerdi et al., 2018; Irvin et al., 2019). The tensile data show that all the MCNC samples had higher tensile strength than the standard. The results of the MCNC binding themselves through -OH create a high strength linkage, with potential interaction among them, thus increasing the adhesive properties of the composite. The increasing MCNCs loading resulted in agglomeration between particles, which weakens the material, explaining the relative decrease in the adhesive properties with individual loading.

### Mechanical Properties

**FIGURE 10:**
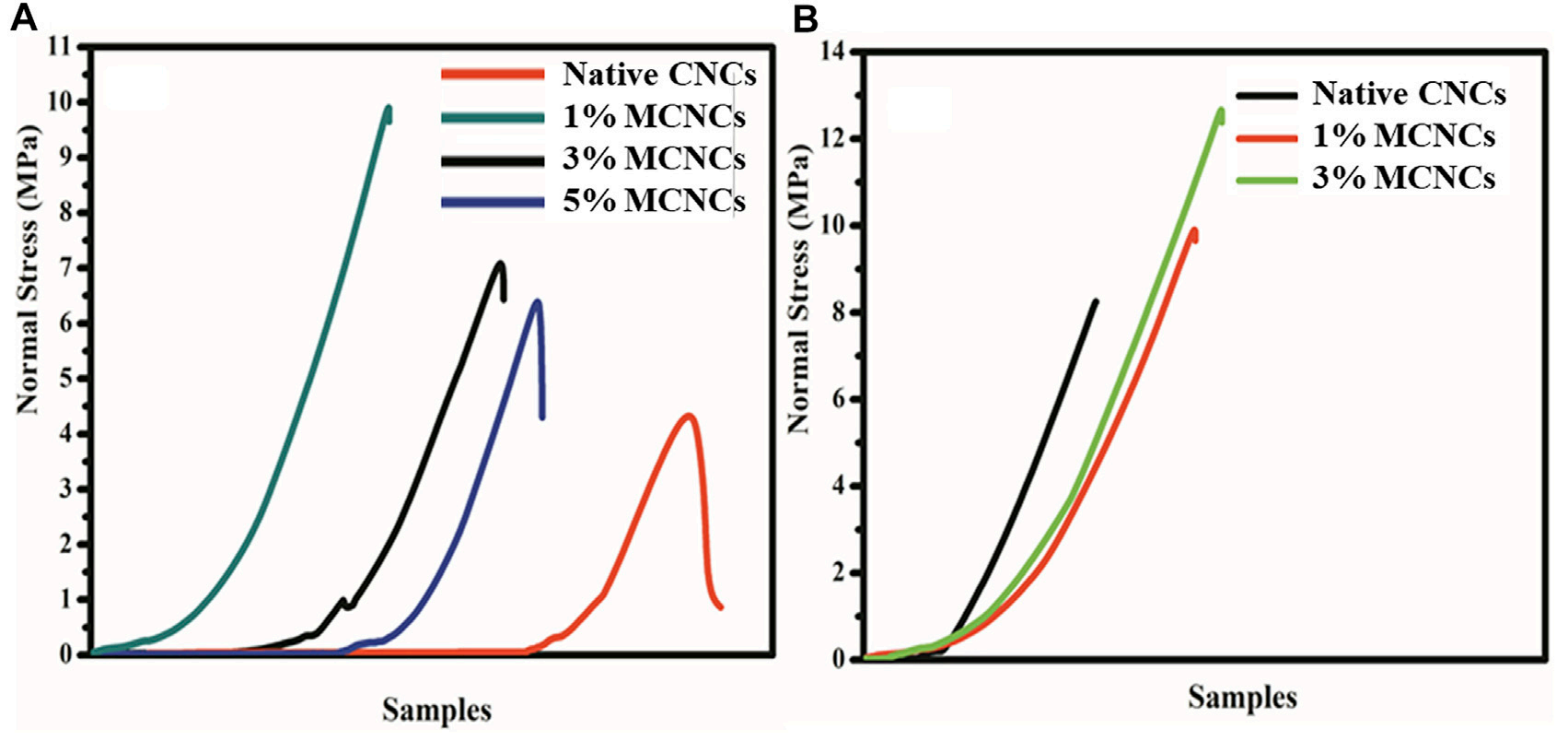
Stress–strain curves of **(A)** standard and different concentrations (1%, 3%, and 5%) of native CNCs and **(B)** standard epoxy and epoxy-modified CNCs (i.e., MCNCs).

The main challenges for using epoxy resins and their composites include the design of high processability (low-viscosity and cured) epoxy with high strength and toughness that can be recycled and reused. Here, we also studied the epoxy system for mechanical properties. The results showed good mechanical properties of MCNCs with the E-51 epoxy system (Ertl, 2021; Zheng et al., 2021). The MCNCs with silane coupling agent showed much improvement, as shown in Figure 10. TETA was used as a curing and toughening agent for thermoset materials for epoxy resins. In recent years, scientists have made significant progress in low synthetic viscosity, such as hyperbranched esterification, etherification, hydrosilylation, polymerization, and oxidation of double bonds. The low viscosity of the curing agent can improve the mechanical properties by separating the entangled molecular chains of the epoxy system. Among the broad applications of TETA, one of the essential uses in the industrial field is their simultaneous reinforcing and toughening function on epoxy. The existing methods of simultaneous reinforcing and toughening of epoxy include the use of nanomaterials, block polymers, and hyperbranched epoxy resins. The homogeneous dispersion and size of nanoparticles and good adhesion between these epoxy and CNCs are critical factors influencing the degree of improvement of strength and toughness (Ahmed et al., 2019; Ahmed et al., 2021).

We faced challenges like highly efficient recycling, understanding the homogeneous reinforcing and toughening mechanism, and sustainable development of thermoset epoxy resins. We prepared degradable hyperbranched epoxy resins by esterification and a thiol-ene reaction based on high-performance inexhaustible based epoxy. The cured epoxy composites showed good mechanical properties and degradability, including high shear stress, high toughness, highly efficient degradation, and recycling, resulting from the combined effects of the hyperbranched topological structure of epoxy and good compatibility.

### The Contact Angle of CNCs With E-51 Epoxy System

**FIGURE 11:**
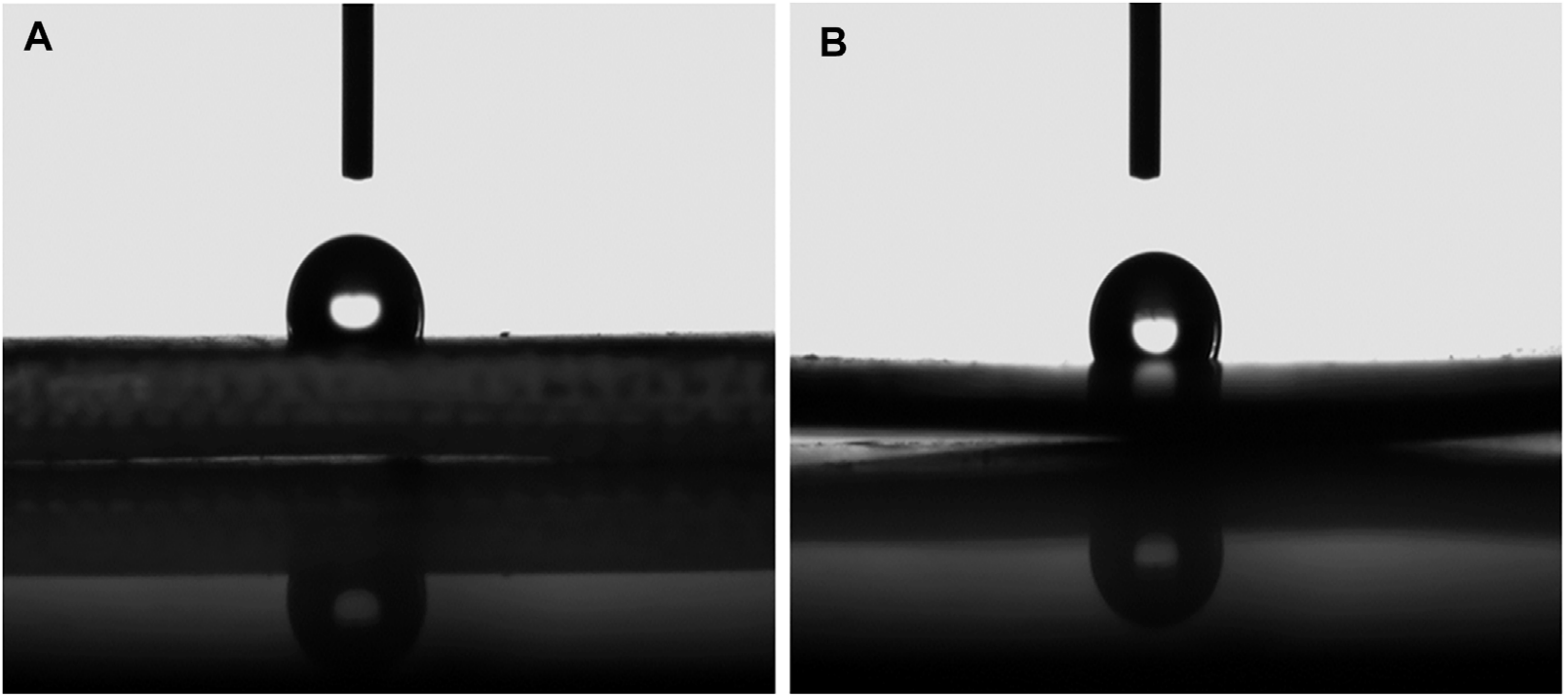
Contact angle measurement of **(A)** native and **(B)** epoxy-modified CNCs (MCNCs).

The inherent properties of hydrophilicity and environmental preferability of CNCs make them great candidates for application in water-treatment membranes. Here, investigations on anisotropic wetting induced by roughness texture after traditional surface generation methodologies such as CNCs milling may be helpful to optimize the selection of machining condition tools. The proposed model can evaluate the wettability of hydrophilic target materials with a non-composite wetting state, incorporating the liquid spreading dynamics, geometrical aspects, and roughness parameters of the solid surface. Considering the practical applications, a verification of the model is presented through systematic experiments. An investigation on the wettability of CNCs with high illumination power monochromatic LED surfaces establishes a hydrophilic, non-composite wetting state during its interaction with water drops. The contact angle is conventionally measured through the liquid, where a liquid–vapor interface meets a solid surface. The contact angle of water on native and modified CNCs with E-51 epoxy were carried out to demonstrate the wettability of native and modified CNCs, as shown in Figure 11. The abundant OH groups of native CNCs are responsible for hydrogen bonding with the water (CA = 60 ± 2°). The native CNCs lost their hydrophilicity after modification with epoxy. The modified CNCs showed hydrophobic behavior (CA = 105 ± 2°) (Ali et al., 2021; Zhang et al., 2021).

## Conclusion

This study synthesized CNCs and then observed their different properties. The matrix of CNCs was successfully modified by 2-carboxyethyl acrylate using a modified approach. CNC matrix modification has been verified through different spectroscopic techniques. MCNCs studied as a reinforcing material for different polymers have been found to have good adhesive performance, achieving high shear strength (15.1 MPa) at 5 wt% and modulus (2,460 MPa) at 1 wt%. 2-carboxyethyl acrylate introduction to CNCs has significant effects on the physicochemical properties of CNCs. MCNCs of adhesive properties are improved due to the dispersion and better interaction between grafted CNCs and the E-51 epoxy system. The mechanical properties were also enhanced and improved. The effects of EBSCA on the adhesive and mechanical properties with epoxy systems indicated that silane-coupling agents could effectively improve the toughness of the epoxy system. CNCs consist of linear polymers without water units, connected to four carbon atoms *via* the β-glycoside bond. Therefore, CNCs play an important role in different industrial applications in this preliminary work.

## Data Availability

The raw data supporting the conclusion of this article will be made available by the authors, without undue reservation.
